# *Candida* infections among neutropenic patients

**Published:** 2016

**Authors:** Rasoul Mohammadi, Elham Foroughifar

**Affiliations:** 1 Department of Medical Parasitology and Mycology, School of Medicine, Infectious Diseases and Tropical Medicine Research Center, Isfahan University of Medical Sciences, Isfahan, Iran; 2 Department of Infectious Diseases, Al-Zahra Hospital, School of Medicine, Isfahan University of Medical Sciences, Isfahan, Iran.

**Keywords:** Candidiasis, Neutropenic patients, Candida species

## Abstract

**Background:**

Systemic candidiasis is a major complication in neutropenic cancer patients undergoing treatment. Most systemic fungal infections emerge from endogenous microflora so the aim of the present study was to identify *Candida* species isolated from the different regions of body in neutropenic patients in compare with the control group.

**Methods:**

A total of 309 neutropenic cancer patients and 584 patients without cancer (control group) entered in the study. Molecular identification of clinical isolates was performed by PCR-RFLP technique.

**Results:**

Twenty-two out of 309 patients had candidiasis (7.1%). Male to female ratio was 1/1 and age ranged from 23 to 66 years. Colorectal cancer and acute myeloid leukemia (AML) were the most common cancers. *Candida albicans* was the most prevalent *Candida *species among neutropenic patients (50%) and control group (57.9%). Mortality rate in cancer patients was 13.6% in comparison with control group (5.2%).

**Conclusion:**

Since candidiasis is an important cause of morbidity and mortality in neutropenic patients, precise identification of *Candida* species by molecular techniques can be useful for the appropriate selection of antifungal drugs particularly in high risk patients.

Systemic candidiasis is an important complication in neutropenic patients and those undergoing treatment for cancer (1). This infection has increased persistently over the past three decades and represents a significant cause of morbidity and mortality among high risk individuals (2). The predisposing factors for systemic candidiasis in neutropenic patients with hematological malignancies differ according to the level of immune suppression and role of the underlying neoplastic process (3, 4). Neutropenia may initiate due to radiation, bone marrow failure (aplastic anemia and myelodysplasia), chemotherapy, and replacement of hematopoietic cells by malignant cells in the bone marrow (3, 5). The digestive tract is the main entrance of *Candida* species in patients with acute neutropenia and leukemia and a region of endogenous microflora. Invasion of *Candida* to bloodstream may occur through disruption of the normal anatomical barriers. *Candida* infections may present as oropharyngeal candidiasis, esophagitis, candidemia, acute or chronic disseminated candidiasis among this population (4, 6, 7). The aim of the present study was to identify *Candida* species isolated from the different regions of body in neutropenic patients in compare with the control group. Due to the different susceptibilities of the conventional antifungal drugs such as fluconazole and itraconazole, timely and precise identification of *Candida* spp. would be noteworthy for successful treatment of the infection.

## Methods

**Isolates: **From March 2014 to August 2015, a total of 309 neutropenic patients with suspected candidiasis from two university hospitals were included in the present study. In addition, we provided a control group without cancer comprised of 584 concurrent hospitalized patients in the ICU (274 patients), transplantation ward (169 patients), and general medicine ward (141 patients) who had no any cancer or cancer history. After sampling, all specimens were examined by direct microscopic examination (DM) with 10% potassium hydroxide (KOH), and culture on sabouraud glucose agar (Difco, Detroit, MI, USA), and CHROMagar Candida (Paris, France). 

**Molecular identification**

**DNA extraction: **The genomic DNA of all isolates was extracted using FTA ® Elute MicroCards (Whatman Inc., Clifton, NJ, USA) (8), following the manufacturer's instructions. Briefly, a loopful of a single colony was suspended in 80-100 μl of distilled water and 5 μl of the suspension was transferred to a disc of FTA card (4 mm in diameter) and incubated at 25°C for at least 5 h. The dried papers were eluted in 400 μl sterile water for 10 seconds, then the paper was transferred to a new microtube containing 40 μl distilled water and incubated at 95 ° C for 15 min. The paper discs were removed and the water including DNA was used for PCR and stored at - 20 °C. 

**Polymerase chain reaction (PCR): **Identification of *Candida* spp. was performed using the already delineated PCR-RFLP profiles (9-11). Briefly, the ITS1-5.8SrDNA-ITS2 region was amplified using PCR mixture including 5μl of 10 × reaction buffer, 0.4 mM dNTPs, 1.5 mM MgCl2, 2.5 U of Taq polymerase, 30 pmol of both ITS1 (5′ -TCC GTA GGT GAA CCT GCG G-3′) and ITS4 (5′ -TCC TCC GCT TAT TGA TAT GC-3′) primers (10), and 2μl of extracted DNA in a final volume of 50μl. The PCR cycling conditions comprised: initial denaturation at 94 ° C for 5 min, followed by 30 cycles of denaturation at 94 °C for 30 s, annealing at 55 °C for 45 s, and extension at 72 ° C for 1 min, with a final extension at 72 °C for 7 min. 

**Restriction fragment length polymorphism (RFLP): **During the second step, PCR products were digested with the restriction enzyme *Hpa*II (Fermentas, Vilnius, Lithuania). 

**Electrophoresis:** Five microliters of each PCR amplicons and 10μl of RFLP products were separated by gel electrophoresis on 1.5 and 2% agarose gel (containing 0.5 μg/ml ethidium bromide), respectively.

**Statistical Analysis: **Data were analyzed using the SPSS software Version 17.0. Prevalence and types of *Candida* infection and their distribution were compared according to sex and age in patients and control group. Chi square and Independent sample t-test were used for analyses. A P-value of < 0.05 was considered significant.

## Results

**Figure 1:**
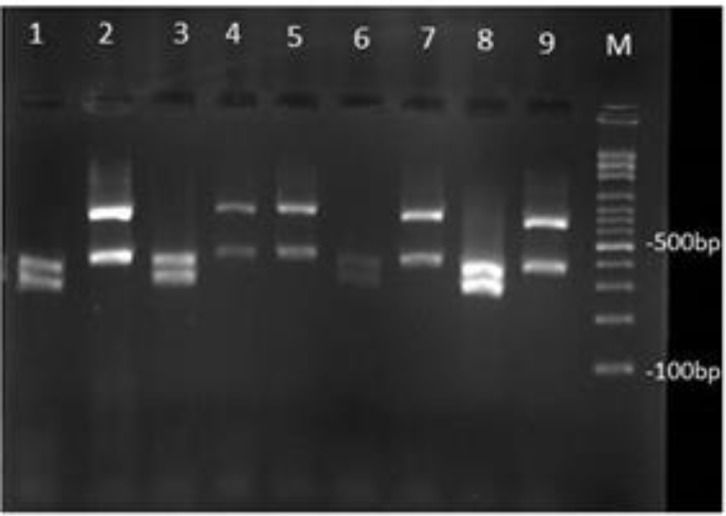
Agarose gel electrophoresis of ITS-PCR products of various *Candida* species after digestion with *Hpa*II. Lanes 1,3,6,8 are *C. albicans*, and Lanes 2, 4, 5, 7, 9 are *C. glabrata*, and Lane M: 100 bp DNA size marker

Twenty-two out of 309 patients had candidiasis (7.1%). Age range of patients was between 23 and 66 years (mean age, 44.5 years). Male to female ratio was 1/1. Colorectal cancer and acute myeloid leukemia (AML) were the most common cancers accounted for 50% of all cases. Cancer patients included 63.6% with organ and 36.4% with hematological malignancies. Clinical specimens were obtained from urine (59.1%), blood (18.2%), skin lesion (13.6%), soft tissue abscess (4.5%), and abdominal abscess (4.5%). The patients had been hospitalized in haematology ward (59.1%), and ICU (40.9%). *Candida albicans* was the most prevalent species (50%) followed by *C. glabrata* (36.3%), and *C. tropicalis* (13.6%) (fig1). 

Table 1 summarizes the characteristics of all study patients. In the control group, 19 out of 584 patients (3.2%) were infected to different forms of candidiasis (Table 2). The mean age of patients in the control group was 35.4 years. In this group, *Candida albicans* was also the most common specie (57.9%) followed by *C. parapsilosis* (21%). There was no case with *C. tropicalis* infection among *Candida *strains isolated from the control group. 

**Table 1:** Details of neutropenic patient with candidiasis

**No**	**Sex**	**Age**	**Hospital wards**	**Alive/ Deceased**	**Cancer of**	**Signs**	**Location body**	**WBC count (/µl)**	**Neutrophil** **(/µl)**	**Neutrophil (%)**	***Candida *** **spp.**
1	F	27	Haematology	Alive	Breast	Breast lumps	Urine	1650	800	48	*C. albicans*
2	M	39	ICU	Alive	AML	Weakness	Blood	2050	760	37	*C. glabrata*
3	M	57	Haematology	Alive	Lung	Cough, Sputum	Urine	2300	950	41	*C. albicans*
4	M	61	Haematology	Alive	Colon	Gastrointestinal bleeding	Urine	1400	670	47	*C. glabrata*
5	F	40	ICU	Alive	Osteosarcoma	Pain in the lower femur	Urine	2700	1150	42	*C. albicans*
6	F	34	ICU	Deceased	AML	Weakness	Blood	900	460	51	*C. albicans*
7	F	42	ICU	Alive	Colon	Gastrointestinal bleeding	Blood	3400	1200	35	*C. albicans*
8	F	61	Haematology	Alive	Colon	Gastrointestinal bleeding	Urine	1400	540	33	*C. albicans*
9	F	54	Haematology	Alive	Breast	Breast lumps	Urine	2150	1200	55	*C. albicans*
10	M	30	Haematology	Alive	Colon	Gastrointestinal bleeding, Constipation	Urine	1300	670	51	*C. glabrata*
11	F	43	Haematology	Alive	Hodgkin's lymphoma	Lymphadenopathy	Skin lesion	2400	1100	45	*C. tropicalis*
12	M	51	ICU	Alive	Colon	Gastrointestinal bleeding	Soft tissue abscess	1080	540	50	*C. glabrata*
13	M	47	ICU	Deceased	Pancreas	Abdominal lumps	Abdominal abscess	1800	920	51	*C. glabrata*
14	M	24	Haematology	Alive	Multiple myeloma	Pain in the bones	Urine	1300	450	34	*C. glabrata*
15	F	31	Haematology	Alive	AML	Weakness	Skin lesion	3100	1050	33	*C. tropicalis*
16	F	54	Haematology	Alive	Stomach	Gastrointestinal bleeding, Abdominal pains	Urine	1700	840	49	*C. albicans*
17	M	23	ICU	Alive	Esophagus	Dysphagia	Urine	2050	900	43	*C. tropicalis*
18	M	62	Haematology	Alive	Colon	Gastrointestinal bleeding	Urine	2700	1100	40	*C. albicans*
19	F	43	ICU	Deceased	AML	Weakness	Blood	1400	650	46	*C. glabrata*
20	M	40	Haematology	Alive	AML	Asymptomatic	Urine	2350	1200	51	*C. albicans*
21	F	66	Haematology	Alive	Hodgkin's lymphoma	Lymphadenopathy, Abdominal pains	Skin lesion	1900	740	38	*C. albicans*
22	M	50	ICU	Alive	Lung	Hemoptysis	Urine	2100	800	38	*C. glabrata*

**Table 2:** Control group in the present study; patients with different forms of candidiasis without cancer

**No**	**Sex**	**Age**	**Hospital wards**	**Alive/ Deceased**	**Clinical site**	**Signs**	**WBC count (/µl)**	**Neutrophil** **(/µl)**	**Neutrophil (%)**	***Candida*** ** spp.**
1	F	5	ICU	Alive	Blood	Fever	15300	10863	71	*C. albicans*
2	F	26	ICU	Alive	Blood	Fever, Pain of joints	13400	9246	69	*C. albicans*
3	F	18	Transplantaion Ward	Alive	Urine	Painful urination	6600	4554	69	*C. parapsilosis*
4	M	55	Transplantaion Ward	Alive	Urine	Fever and chills	8100	3969	49	*C. albicans*
5	F	63	ICU	Alive	Blood	Fever and chills	16900	9800	58	*C. albicans*
6	F	49	ICU	Deceased	Blood	Fever	19400	15520	80	*C. albicans*
7	F	38	General ward	Alive	Vulvovagina	Vulvovaginal discharge	10500	5670	54	*C. parapsilosis*
8	F	11	Transplantaion Ward	Alive	Blood	Pain and tenderness	9100	6825	75	*C. albicans*
9	M	27	ICU	Alive	Blood	Fever	14000	9940	71	*C. albicans*
10	M	39	ICU	Alive	Skin lesion	Inflammatory, Pruritus	8200	5330	65	*C. parapsilosis*
11	F	41	Transplantaion Ward	Alive	Urine	Fever and chills	9450	6140	65	*C. albicans*
12	M	17	ICU	Alive	Catheter	Fever	21000	11130	53	*C. albicans*
13	M	14	ICU	Alive	Blood	Fever	11700	8892	76	*C. albicans*
14	M	55	ICU	Alive	BAL	Cough, Chest pain	11050	6630	60	*C. krusei*
15	F	69	Transplantaion Ward	Alive	Blood	Fever	14900	10280	69	*C. albicans*
16	M	27	General ward	Alive	Urine	Fever	8800	5016	57	*C. kefyr*
17	F	20	General ward	Alive	Skin lesion	Pruritus	7600	5320	70	*C. glabrata*
18	F	48	General ward	Alive	Urine	Asymptomatic	12650	7843	62	*C. parapsilosis*
19	F	51	ICU	Alive	Perleche	Pruritus	6550	4322	65	*C. glabrata*

Twelve patients (63.1%) were females and 7 control patients (36.8%) were males, age ranging from 5 to 69 years. Surprisingly, all *Candida* species that were isolated from blood stream were *C. albicans*. Mortality rate in cancer patients (13.6%) was significantly higher than the control group (5.2%). 

*Candida* infection in cancer patients was greater than the control group [OR (CI 95%): 2.28 (1.21-4.28%), P=0.009] (table 3).

**Table 3:** Statistical analysis of candidosis among neutropenic patients and control group

**Factors**	**Cancer(n=309)**	**Control(n=584)**	**P value**
Age(year)	44.50±12.91	35.42±18.83	0.076^□^
**Sex** Male Female	11(50.0%) 11(50.0%)	7(36.8%) 12(63.2%)	0.397□
**Candidiasis** Yes No	22(7.1%) 287(92.9%)	19(3.3%) 565(96.7%)	0.009□

Data Showed Mean±SD and n(%),: Used of Independent sample t test, : Used of Chi-Square

## Discussion

Most fatal *Candida *infections result from endogenous host microbiota (9, 10). Colonization due to the non-*Candida albicans* spp. is increasing (2, 11, 12), and in recent years significant increase in frequency of blood stream isolated infection has been reported in particular *Candida* infection due to *C. krusei, C. tropicalis* and *C. glabrata* in high risk population, like patients with neutropenia is of serious concern. In the present study, we also isolated 2 out of 4 (50%) *C. glabrata* from cancer patients with candidemia. 

However, no *C. glabrata* strain was isolated from the bloodstream infection in the control group. The intestinal tract is the main source for hematogenous *Candida* invasion (13-15). Mortality rate was 13.6% and 5.2% in neutropenic patients and control group, respectively. As expected, mortality rate in patients with candidemia was the highest in both groups. There has been a crucial shift in the causes of blood stream *Candida* infection from *C. albicans* toward non-*albicans Candida* species in neutropenic patients (4), but *C. albicans* was the most prevalent strain isolated from candidemia in the control group. Candidemia in neutropenic patients may be complicated by chronic disseminated candidiasis of eyes, spleen, liver, kidney, and abdomen (16). We also showed two patients (9.1%) with soft tissue abscess, and abdominal abscess as a result of chronic disseminated candidiasis. 

Among the patients with candiduria, 7 patients (53.8%) had lower urinary tract symptoms (LUTS) (such as painful urination, increased frequency of urination, and incomplete voiding), 2 patients (15.4%) had upper urinary tract symptoms (UUTS) (including fever, chills, pain and tenderness, nausea, and vomiting), and 4 (30.7%) cases were asymptomatic, compared to the control group that 2 patients (40%) had UUTS, 2 patients (40%) with LUTS, and 1 patient (20%) was asymptomatic. 

The prevalence of candiduria is associated with antibiotic use (17), and varies in different hospital wards, being most prevalent in intensive care units (ICUs) (18) however, in the present study, only two patients with candiduria were hospitalized in ICU (in cancer patients) and also none of the patients in control group with candiduria hospitalized in ICU. Some studies showed that a low percentage (1-8%) of candiduric patients presents candidemia (19-21), however patients with candiduria in the present investigation did not shift toward bloodstream *Candida* infection except a patient undergoing kidney transplantation in the control group. In contrast to our findings, in many investigations *C. parapsilosis* complex was the main *Candida* species that is associated with candidiasis, containing candiduria (22-24), nevertheless, we did not isolate any *C. parapsilosis* complex from neutropenic patient whereas, 4 cases of *C. parapsilosis* (21%) isolates were identified in the control group. Afraseyabi et al. (25) isolated 19 *Candida* spp. from 60 cancer patients (31.6%). They reported gastrointestinal and breast cancer as the most frequent cancer whereas, colorectal cancer and acute myeloid leukemia (AML) were the most common cancers in the present study. Shokohi et al. (26) reported *Candida albicans* as the most common species among 80 neutropenic patients with candidosis (77.5%), followed by *C.glabrata* (15%), *C. tropicalis* (5%) and *C. krusei* (2.5%). Saltanatpouri et al. (27) reported *C.albicans* as the most prevalent *Candida* strain isolated from candidiasis in cancer patients. Brain tumor and esophageal cancer were the most frequent cancers in their investigation. Of the 68 blood samples collected from cancer patients, Kalantar et al. (28) showed that five (7.35%) were positive for *Candida* spp., 2 (40%) of which were identified as *C. albicans* and 3 (60%) were *Candida* non-*albicans*.

In conclusion, neutropenic population which has noticeable colonization with *Candida* spp particularly in different parts of the body and presence of *C. glabrata*, *C. tropicalis* or *C. krusei* should be considered as higher risk of mortality. Administration of fluconazole seems to be reasonable in preventing candidiasis due to *C. albicans* in neutopenic patients, but strategies to decrease *Candida* infections by nontriazole susceptible to *Candida *species like *C. glabrata* are unreliable. Due to the fact that candidiasis is connected with high morbidity and mortality rate among neutropenic patients, and emerging of antifungal resistance among *Candida* isolates, epidemiological data and susceptibility patterns of colonized *Candida* species may be useful for clinicians to select the best therapeutic choice for the management of infection among high-risk cases. 

## Notes

**Funding: **This study was not supported financially by any governmental or other public sources.
